# Supplementary material for: Causal interplay between lactose intolerance and gut microbiota: a combined bidirectional Mendelian randomization and in vivo validation study
Source: Front Nutr. 2026 Jun 1;13:1803337. doi: 10.3389/fnut.2026.1803337 (PMC13265576; doi:10.3389/fnut.2026.1803337)
Supplement: Supplementary file 1 [file Data_Sheet_1.zip › supplementary materials/Forward/leave one out cross validation/ebi-a-GCST90027503.finngen_R12_E4_LACTONAS.pdf]

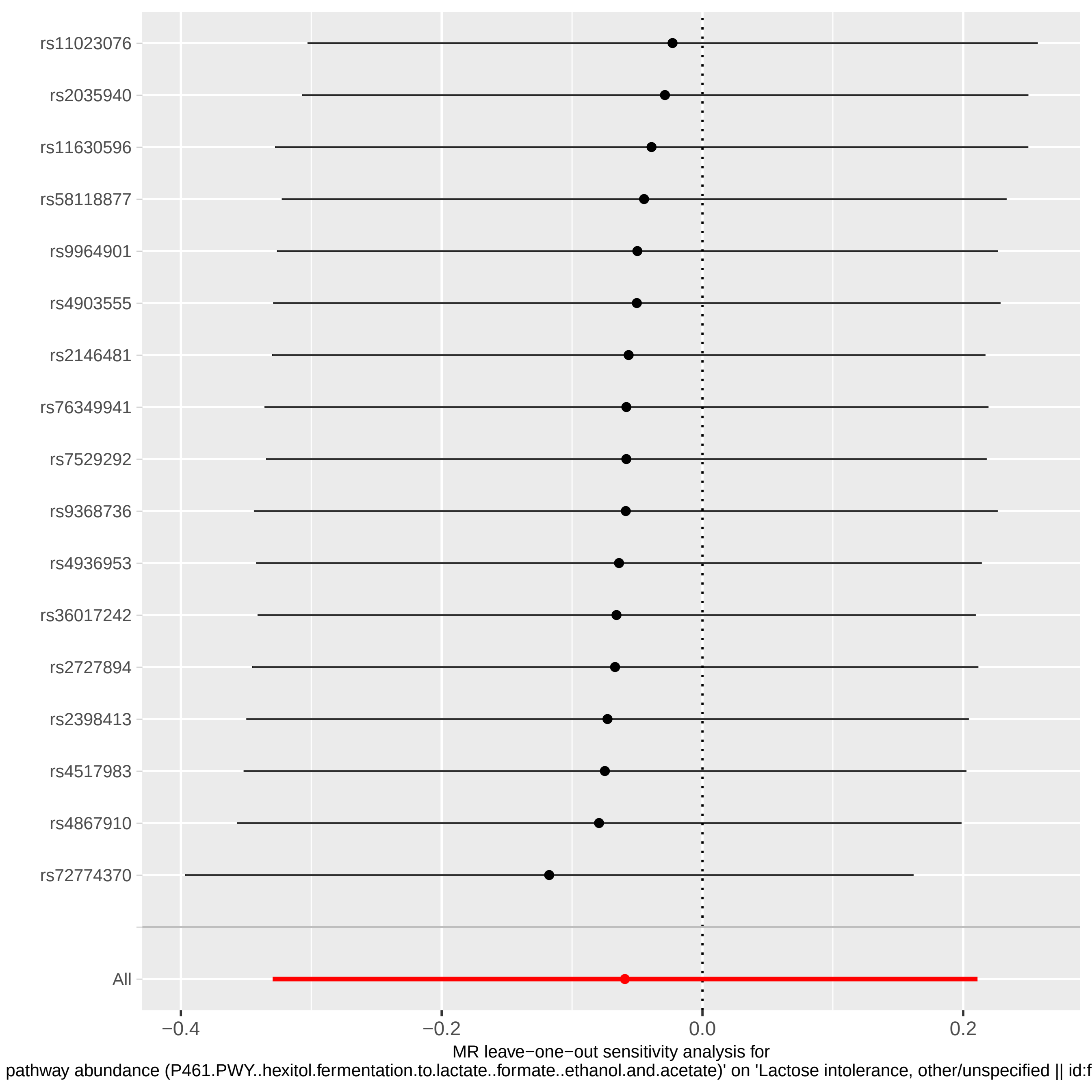
rs11023076
rs2035940
rs11630596
rs58118877
rs9964901
rs4903555
rs2146481
rs76349941
rs7529292
rs9368736
rs4936953
rs36017242
rs2727894
rs2398413
rs4517983
rs4867910
rs72774370
All
−0.4
−0.2
0.0
0.2
MR leave−one−out sensitivity analysis for
pathway abundance (P461.PWY..hexitol.fermentation.to.lactate..formate..ethanol.and.acetate)' on 'Lactose intolerance, other/unspecified || id:f
